# Polybrominated Diphenyl Ether (PBDE) Flame Retardants and Thyroid Hormone during Pregnancy

**DOI:** 10.1289/ehp.1001905

**Published:** 2010-06-21

**Authors:** Jonathan Chevrier, Kim G. Harley, Asa Bradman, Myriam Gharbi, Andreas Sjödin, Brenda Eskenazi

**Affiliations:** 1 Center for Children’s Environmental Health Research, School of Public Health, University of California–Berkeley, Berkeley, California, USA; 2 Pôle d’Épidémiologie et Santé Publique, Institut Pasteur, Paris, France; 3 Division for Laboratory Sciences, National Center for Environmental Health, Centers for Disease Control and Prevention, Atlanta, Georgia, USA

**Keywords:** endocrine disruption, flame retardants, persistent organic pollutants, polybrominated diphenyl ethers (PBDEs), pregnancy, thyroid hormone

## Abstract

**Background:**

Human exposure to polybrominated diphenyl ether (PBDE) flame retardants has increased exponentially over the last three decades. Animal and human studies suggest that PBDEs may disrupt thyroid function. Although thyroid hormone (TH) of maternal origin plays an essential role in normal fetal brain development, there is a paucity of human data regarding associations between exposure to PBDEs and maternal TH levels during pregnancy.

**Objectives:**

Our goal was to determine whether PBDE serum concentrations are associated with TH levels in pregnant women.

**Methods:**

We measured the concentration of 10 PBDE congeners, free thyroxine (T_4_), total T_4,_ and thyroid-stimulating hormone (TSH) in 270 pregnant women around the 27th week of gestation.

**Results:**

Serum concentrations of individual PBDE congeners with detection frequencies > 50% (BDEs 28, 47, 99, 100, and 153) and their sum (∑PBDEs) were inversely associated with TSH levels. Decreases in TSH ranged between 10.9% [95% confidence interval (CI), −20.6 to 0.0] and 18.7% (95% CI, −29.2 to −4.5) for every 10-fold increase in the concentration of individual congeners. Odds of subclinical hyperthyroidism (low TSH but normal T_4_) were also significantly elevated in participants in the highest quartile of ∑PBDEs and BDEs 100 and 153 relative to those in the first quartile. Associations between PBDEs and free and total T_4_ were not statistically significant. Results were not substantially altered after the removal of outliers and were independent of the method used to adjust for blood lipid levels and to express ∑PBDEs.

**Conclusions:**

Results suggest that exposure to PBDEs is associated with lower TSH during pregnancy. Findings may have implications for maternal health and fetal development.

Polybrominated diphenyl ethers (PBDEs) are synthetic chemicals used as flame retardants in a variety of consumer products such as electronics, furniture, textiles, and construction materials. The chemical structure and properties of PBDEs are similar to those of polychlorinated biphenyls (PCBs), which were banned in the United States in 1978 (Toxic Substances Control Act of 1976). Theoretically, a total of 209 PBDE congeners may be produced depending on the number and position of bromine atoms on the diphenyl ether structure, although only congeners with more than four bromines are used commercially. PBDEs are lipophilic, bioaccumulate in wildlife and humans, and biomagnify up the food chain (Burreau et al. 1999). Congeners with lower bromine contents are particularly persistent, with estimated half-lives ranging between 2 and 12 years in humans (Geyer et al. 2004). PBDEs are global contaminants that have been detected in human adipose tissue, serum, and/or breast milk samples collected in Asia, Europe, North America, Oceania, and the Arctic (Bi et al. 2006; Harrad and Porter 2007; Pereg et al. 2003; Sjodin et al. 1999, 2008). The concentration of these chemicals in human serum and breast milk has exponentially increased in the last three decades (Noren and Meironyte 2000; Schecter et al. 2005; Sjodin et al. 2004a).

Prenatal exposure to PBDEs has been reported to alter spontaneous motor behavior, memory, and learning in rats and mice (Kuriyama et al. 2005; Viberg et al. 2003). In humans, higher maternal serum PBDE levels were found to be related with lower scores on measures of intelligence and attention (Herbstman et al. 2010; Roze et al. 2009) and increased time to pregnancy (Harley et al. 2010). Maternal thyroid hormones (THs) play an essential role in fetal brain development (Auso et al. 2004; Haddow et al. 1999) and modulate menstrual cycle characteristics (Poppe and Velkeniers 2004). It has thus been suggested that PBDEs may affect neurodevelopment and fertility by disrupting THs (Agency for Toxic Substances and Disease Registry 2004; Harley et al. 2010).

Similarly to studies on PCBs (Craft et al. 2002; Desaulniers et al. 1999), most studies conducted in nonpregnant rats and mice report that exposure to PBDEs lowers free and/or total thyroxine (T_4_) in a dose-dependent fashion and thus has a hypothyroxinemic effect; PBDE exposure generally did not affect thyroid-stimulating hormone (TSH) levels [see Supplemental Material, Table 1a (doi:10.1289/ehp.0901905)] (Hallgren and Darnerud 2002; Hallgren et al. 2001; Zhou et al. 2001). Only two experimental studies have been conducted in pregnant animals. Zhou et al. (2002) exposed rat dams daily to the PBDE commercial mixture DE-71 at doses of 0, 1, 10, and 30 mg/kg from gestation day (GD) 6 to postnatal day (PND) 21 and found a 48% and 44% decrease in total T_4_ in the high-exposure group relative to controls at GD20 and PND22, respectively (free T_4_ not measured). Skarman et al. (2005), however, did not find daily exposure to PBDE congener BDE-99 or the commercial mixture Bromkal 70-5DE beginning on GD4 to affect total or free T_4_ levels in mice at GD17.

In contrast to animal experimental studies, human epidemiologic studies conducted in nonpregnant adults generally reported lower TSH and higher free and total T_4_ in relation with higher PBDE concentrations in serum (Bloom et al. 2008; Dallaire et al. 2009; Hagmar et al. 2001; Julander et al. 2005; Turyk et al. 2008) and house dust (Meeker et al. 2009), suggesting that these chemicals may exert a hyperthyroidic effect [see Supplemental Material, Table 1b (doi:10.1289/ehp.1001905)]. Turyk et al. (2008), for instance, found positive associations between the sum of eight PBDE congeners and both free and total T_4_, and an inverse association with TSH in 308 male Great Lakes fish consumers. Participants in the fourth quartile of exposure to BDEs 99 and 153, but not BDEs 47 and 100, had modestly increased free and total T_4_ levels relative to those in the first quartile of exposure. In addition, odds of hyperthyroidism were 5.7 times higher [95% confidence interval (CI), 0.9–36.4] in men with total PBDEs (∑PBDEs) above versus below the 90th percentile (0.78 ng/g serum). In the largest study to date (*n* = 623), Dallaire et al. (2009) found a positive relation between plasma BDE-153, but not BDE-47, and total triiodothyronine (T_3_) in Inuits; they also reported nonstatistically significant inverse relationships between BDEs 47 and 153 and TSH. Contrary to the above studies, Yuan et al. (2008) found higher serum TSH in 23 highly exposed (median ∑PBDEs, 382 ng/g lipids) Chinese electronic-waste workers relative to 26 controls who also had elevated serum ∑PBDE concentrations (median, 158 ng/g lipids).

Only one small study (*n* = 9) examined associations between serum PBDE and TH concentrations in women during pregnancy; no associations were found between ∑PBDEs and free or total T_4_ in serum samples collected shortly before delivery (Mazdai et al. 2003). Herbstman et al. (2008) measured PBDEs and TH in cord serum and reported that higher concentrations of BDE-100, but not BDEs 47 and 153, were associated with lower (< 20th percentile) total T_4_ [odds ratio (OR) = 2.1; 95% CI, 1.1–4.2] among women who had spontaneous unassisted vaginal deliveries only (*n* = 92). BDEs 100 and 47, but not BDE-153, were associated with a reduced likelihood of high (> 80th percentile) TSH levels (OR = 0.4; 95% CI, 0.2–0.8 for both chemicals) among the same women.

We have previously reported a positive association between PCBs grouped according to their potential to induce uridine diphosphate glucuronosyltransferase (UDP-GT) in rodents and neonatal TSH (Chevrier et al. 2007) and between total PCBs and free T_4_ in pregnant women (Chevrier et al. 2008). The purpose of the present investigation was to determine the relation between serum PBDE concentrations and thyroid function in the same population of pregnant low-income Latina women living in California.

## Materials and Methods

### Participants

Data from the Center for the Health Assessment of Mothers and Children of Salinas (CHAMACOS), a birth cohort study of health and environmental exposures, were used for this study. Pregnant women who sought prenatal care at one of six participating health clinics between October 1999 and October 2000 and were < 20 weeks gestation, ≥ 18 years of age, eligible for state-sponsored health care (Medi-Cal), and intended to deliver at Natividad Medical Center (Monterey County, CA, USA) were enrolled in CHAMACOS (*n* = 601). Women were excluded from the present analyses if they did not participate through delivery or did not give birth to a live child (*n* = 64), bore twins (*n* = 5), refused to give a blood sample or gave a sample of insufficient volume for PBDE (*n* = 168) or TH (*n* = 69) analyses, or took medication that could affect TH levels (*n* = 1). In addition, data were not reported for 24 serum samples that failed to meet quality assurance standards for PBDE measurement. A total of 270 women were thus included in this analysis. Study participants provided written informed consent, and all research activities were approved by the University of California–Berkeley Committee for the Protection of Human Subjects.

### Data collection

Participants were interviewed at enrollment (mean ± SD, 14.0 ± 5.0 weeks gestation) and at the end of the second trimester of pregnancy (26.5 ± 2.4 weeks gestation) using structured questionnaires. Information about demographics, country of birth, time lived in the United States, parity, and health-related behaviors was collected. Medical records were abstracted by a registered nurse to obtain data on thyroid-related diseases, medication use, and general health status.

Blood samples were collected around the time of the second interview (mean ± SD, 27.3 ± 3.1 weeks’ gestation) and were immediately processed and stored at −80°C. PBDEs were measured in serum at the Centers for Disease Control and Prevention (Atlanta, GA, USA) using gas chromatography/isotope-dilution high-resolution mass spectrometry (GC-IDHRMS) (Sjodin et al. 2004b). PBDE concentrations are expressed on a blood lipid basis. Total lipids were determined based on the measurement of triglyceride and total cholesterol in serum using standard enzymatic methods (Roche Chemicals, Indianapolis, IN, USA) (Phillips et al. 1989). Limits of detection (LODs) ranged between 0.2 and 1.6 ng/g lipids except for BDE-47 (range, 0.8–5.6 ng/g lipids). Quality control samples were included in each run. THs were measured in serum by Quest Diagnostics’ Nichols Institute (San Juan Capistrano, CA, USA). Free T_4_ was measured by direct equilibrium dialysis followed by radioimmunoassay (Nelson and Tomei 1988). Although results obtained by commonly used immunoassays may be affected by the blood concentration of T_4_-bound proteins (Wang et al. 2000), which increases during pregnancy (Glinoer 1997), equilibrium dialysis physically separates the free from the bound hormone before measuring it with a highly sensitive immunoassay. This method yields accurate measurements in samples with normal and elevated T_4_-bound protein concentrations (Nelson et al. 1994). Total T_4_ was measured by solid-phase immunochemiluminometric assay, and TSH by ultrasensitive third-generation immunochemiluminometric assay. LODs were 0.1 ng/dL, 0.1 μg/dL, and 0.01 mIU/L for free T_4_, total T_4_, and TSH, respectively.

To control for other environmental exposures that may affect TH, we measured PCBs and organochlorine pesticides [including hexachlorobenzene (HCB), *p*,*p*′-dichlorodiphenyl trichloroethane (DDT), *o*,*p*′-DDT, *p*,*p*′-dichlorodiphenyl dichloroethylene (DDE), γ-hexachlorocyclohexane, dieldrin, mirex, and *trans*-nonachlor] in serum samples using GC-IDHRMS, and lead in a subset of maternal (*n* = 70) and umbilical cord (*n* = 161) blood samples using graphite furnace atomic absorption spectrophotometry. PCBs and organochlorine pesticides were expressed on a serum lipid basis.

### Statistical analysis

We log_10_-transformed the serum concentrations of PBDE congeners to reduce the effect of outliers. We used Pearson’s correlations to evaluate the interrelationship of PBDE congeners and analysis of variance (ANOVA) to examine associations between demographic characteristics and PBDE serum concentrations. We used multiple linear regression models to investigate the relationship between maternal PBDE and TH serum concentrations. Free and total T_4_ were normally distributed, whereas TSH was right-skewed and was thus log_10_-transformed to approximate a normal distribution. We ran separate models for each congener with a detection frequency > 50% (BDEs 28, 47, 99, 100, and 153) and for their sum. We ran models expressing exposure continuously and also categorically for each quartile of PBDE to investigate the possibility of a threshold for effect or other nonmonotonic exposure–response relationship. In addition, we fit generalized additive models with 3-degrees-of-freedom cubic splines to evaluate the shape of exposure–response curves and to test for digression from linearity while controlling for covariates. Using multiple logistic regression, we investigated associations between PBDE serum concentrations and maternal hyperthyroidism based on laboratory reference ranges for women in their second (TSH < 0.5 mIU/L) or third (TSH < 0.8 mIU/L) trimester of pregnancy.

Potential confounders considered for inclusion in models (categorized as shown in Table 1 or expressed as indicated in parentheses) comprised maternal age (continuously), race/ethnicity, education, family income, country of birth, number of years spent in the United States, parity, body mass index, gestational age at the time of blood collection (in weeks, continuously), and smoking, alcohol, and drug consumption during pregnancy. We also considered environmental exposures such as blood lead, serum PCB [the sum of congeners with detection frequencies > 75% and of potential UDP-GT inducers (Chevrier et al. 2007)], and organochlorine pesticide concentrations, and we examined the possibility of an interaction between PBDEs (continuously) and PCBs (continuously, and dichotomized at the 75th and 90th percentiles), which was previously reported in rats (Hallgren and Darnerud 2002). All environmental exposures were log_10_-transformed. We had complete data on most covariates. Values of missing covariates were imputed at random based on observed probability distributions (< 2% missing) or on prediction models using nonmissing variables (≥ 2% missing). Covariates associated with any of the outcomes (*p* < 0.20) were included in all models. Final models comprised maternal age at enrollment, education, country of birth, gestational age at the time of blood collection, and family income as well as maternal HCB and total PCB serum concentrations.

Signals below instruments’ LODs may yield better estimates of true concentrations than imputed values. When possible, we thus used values < LOD as measured by instruments. Undetected values were imputed based on a log-normal probability distribution whose parameters were estimated by maximum likelihood estimation. This procedure has been reported to perform better than simple substitution methods using LOD/2 or 
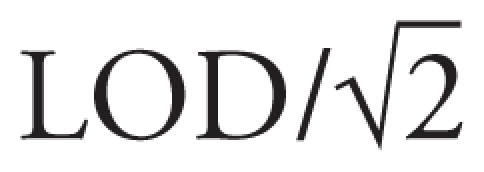
 (Baccarelli et al. 2005; Helsel 1990, 2005; Lubin et al. 2004). Undetected TSH levels were assigned a value equal to half the LOD of 0.01 mIU/L.

We conducted sensitivity analysis to evaluate the robustness of our results. We re-ran models excluding outliers with externally studentized residuals > 3. We also applied separate models expressing PBDEs on a total lipid basis, and on a serum basis while including triglycerides and total cholesterol as covariates in models, and by expressing ∑PBDEs on a weight and molar basis. In addition, we ran models with untransformed PBDE values and outliers excluded.

Because findings were similar for all the models described above, we present only results from regressions with potential outliers included, log_10_-transformed exposures expressed on a serum lipid basis (nanograms per gram lipids), and ∑PBDEs as well as individual congeners expressed on a weight basis. Statistical significance was set at *p* < 0.05 for main effects and *p* < 0.10 for interactions based on two-sided tests. Statistical analyses were performed using STATA/IC (version 10.1; StataCorp LP, College Station, TX, USA) and R (version 2.7.1; R Foundation for Statistical Computing, Vienna, Austria).

## Results

### Population characteristics

Participants were mostly young (mean ± SD, 25.5 ± 5.0 years), low income (62% ≤ federal poverty threshold), Latina (95%) women who had emigrated from Mexico within 10 years at the time of enrollment (75%; Table 1). Most women were multiparous (67%) and did not have a high school diploma (77%). Although a small percentage of women reported that they smoked (7%) or used illegal drugs (2%), almost a quarter (24%) consumed some alcohol during pregnancy, and most (61%) were overweight or obese before pregnancy.

### PBDE concentrations

Table 2 shows the serum concentrations and detection frequencies of individual PBDE congeners and of the sum of congeners with detection frequencies > 50% (∑PBDEs). PBDE levels were lower than those reported in a nationally representative sample of nonpregnant adults (Sjodin et al. 2008). However, levels in CHAMACOS women were likely higher before pregnancy because changes in fat mass alter the serum concentration of persistent organic pollutants (Chevrier et al. 2000). As reported in previous studies, BDEs 47, 99, 100, and 153 had the highest detection frequencies (> 98% detection) and accounted for virtually all ∑PBDEs. BDE-47 contributed more than half of ∑PBDEs, followed by BDEs 99, 153, and 100. We detected BDEs 17, 66, 85, 154, and 183 in < 50% of samples and did not consider them in further analyses. PBDE congeners were moderately to strongly intercorrelated (*r* = 0.6–0.9, *p* < 0.001); BDEs 47, 99, and 100, which are the main components of penta-BDE commercial mixtures, were strongly intercorrelated [*r* > 0.9, *p* < 0.001; see Supplemental Material, Table 2 (doi:10.1289/ehp.1001905)].

### TH concentrations

Mean (± SD) serum concentrations of free and total T_4_ were 0.83 ± 0.24 ng/dL and 10.7 ± 1.6 μg/dL, respectively; the geometric mean for TSH was 1.2 (geometric SD = 1.7) mIU/L. Six participants had low free T_4_ (< 0.5 ng/dL), 10 had low total T_4_ (< 8.0 μg/dL), and one had high TSH concentrations (> 4.6 and > 5.2 mIU/L in second and third trimesters, respectively) based on laboratory reference ranges; TSH was elevated in 14 women based on National Academy of Clinical Biochemistry guidelines (> 2.5 mIU/L) (Mandel et al. 2005). TSH was suppressed (< 0.01 mIU/L) in two women and was low (< 0.5 and < 0.8 mIU/L in second and third trimesters, respectively) in 35 women; four women had high free T_4_ (> 1.6 ng/dL), whereas none had elevated total T_4_ (> 17.8 and > 20.1 μg/dL in second and third trimesters, respectively). Free and total T_4_ decreased with age in a linear fashion (*r* = −0.21 and −0.25, respectively; *p* < 0.001); TSH was not associated with age. Other demographic characteristics were not significantly associated with TH levels (data not shown).

### Associations between PBDE and TH serum concentrations

**Figure 1:**
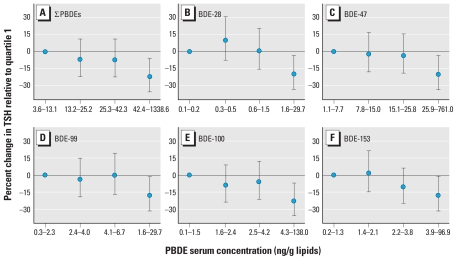
Percent change in geometric mean TSH by quartile of serum PBDE concentration in pregnant women participating in the CHAMACOS study: ∑PBDEs (*A*) and BDEs 28 (*B*), 47 (*C*), 99 (*D*), 100 (*E*), and 153 (*F*). Results are based on multiple linear regression models adjusted for maternal age at enrollment, education, country of birth, gestational age at the time of blood collection, and family income as well as maternal serum concentrations of HCB and ∑PCB. Error bars indicate 95% CIs.

Table 3 shows that none of the PBDE congeners were significantly associated with free or total T_4_ concentrations. Despite small coefficients of determination (*R*^2^), all PBDE congeners were significantly inversely associated with TSH. Associations ranged between a 10.9% (95% CI, −20.6 to 0.0%) and a 18.7% (95% CI, −29.2 to −4.5%) decrease in TSH for every 10-fold increase in the serum concentration of individual congeners (computed from Table 3). A 10-fold increase in ∑PBDE was associated with a 16.8% (95% CI, −27.6 to −2.3%) decrease in TSH, corresponding to a 37.7% decrement over the full range of ∑PBDEs. Furthermore, although tests for digression from linearity were not statistically significant after the exclusion of the two participants with suppressed TSH (data not shown), categorizing PBDEs into quartiles provided some evidence suggestive of nonmonotonic exposure–response relationships (Figure 1).

Given these results, we also investigated associations between PBDE serum concentrations and maternal hyperthyroidism using laboratory reference ranges for women in their second or third trimester of pregnancy. Clinical hyperthyroidism is characterized by depressed TSH and elevated free T_4_. Except in one case, all women with low TSH in this population had normal free T_4_ levels, corresponding to the definition of subclinical hyperthyroidism (Surks et al. 2004). Odds of subclinical hyperthyroidism were nonsignificantly increased 1.9 times (95% CI, 0.8–4.5) for each 10-fold increase in ∑PBDEs and ranged between 1.6 (95% CI, 0.7–3.7) for BDEs 99 and 2.4 (95% CI, 0.9–6.1) for BDE-153 (Table 4). Women in the highest quartile of ∑PBDEs and BDEs 100 and 153 had significantly increased odds of subclinical hyperthyroidism relative to women in the first quartile.

The serum concentration of other chemicals did not confound associations, and in contrast to results observed in rats (Hallgren and Darnerud 2002), we found no effect modification by PCBs (medians = 65.4 ng/g lipids for ∑PCBs and 19.6 ng/g lipids for enzyme inducers; data not shown).

## Discussion

We report significant inverse associations between TSH concentrations and serum measurements of ∑PBDEs and BDEs 28, 47, 99, 100, and 153 in pregnant women. The odds of subclinical hyperthyroidism were also elevated in relation to ∑PBDEs and BDEs 100 and 153. Associations appeared to be primarily due to a decrease in TSH in participants in the highest quartile of PBDE serum concentrations. Relationships between ∑PBDEs and individual PBDE congeners and free T_4_ were generally null, and associations with total T_4_ were mostly inverse, but none were statistically significant.

This is the largest study to investigate associations between PBDEs and TH serum concentrations in pregnant women. Only one small study (*n* = 9) previously examined the question and found no association between the sum of BDEs 47, 99, 100, 153, 154, and 183 and free or total T_4_ but did not measure TSH (Mazdai et al. 2003). Contrary to most studies conducted in nonpregnant adults (Bloom et al. 2008; Dallaire et al. 2009; Turyk et al. 2008), we did not find positive trends between PBDE exposure and free T_4_. Although this discrepancy may be explained in part by differences in the methods used to measure free T_4_ by prior studies (immunoassays) relative to the present study (direct equilibrium dialysis), elevated free T_4_ suggests that exposure to PBDE may have a hyperthyroidic effect, which is consistent with our results of decreased TSH. For the most part, previous studies of nonpregnant adults did suggest reduced TSH serum concentrations in relation to higher PBDE exposure (Bloom et al. 2008; Dallaire et al. 2009; Hagmar et al. 2001; Turyk et al. 2008). Lending support to our results, a recent study (Turyk et al. 2008) found that men with ∑PBDEs > 95th percentile (193 ng/g lipids) had substantially increased odds of having detectable serum thyroglobulin antibodies (OR = 6.1; 95% CI, 1.9–19.2), which are found in 80% of Graves disease patients (Weetman 2000). Graves disease is believed to be the major cause of hyperthyroidism during pregnancy, accounting for > 85% of cases, and may play a role in subclinical hyperthyroidism (Glinoer 1997; Mestman 1997).

It is unclear whether low maternal TSH affects fetal health because *in vitro* studies suggest that human placental permeability to TSH is limited (Bajoria and Fisk 1998). Only one study has investigated the relation between maternal subclinical hyperthyroidism and adverse pregnancy outcomes in humans (Casey et al. 2006). The authors found no increase in low birth weight, major malformations, or fetal, neonatal, or perinatal mortality in infants of 433 women with TSH levels ≤ 2.5th percentile for gestational age and nonelevated free T_4_ levels (≤ 1.75 ng/dL) relative to 23,124 women with normal TSH levels. Nevertheless, subclinical hyperthyroidism may lead to clinical hyperthyroidism (Surks et al. 2004), and hyperthyroidism during pregnancy has been linked with increased risks of miscarriage, premature birth, and intrauterine growth retardation (Lazarus 2005). No studies have investigated the latent effects on subsequent child health or development. Evans et al. (2002), however, reported that brain neuronal and glial cell differentiation is affected in offspring of partially thyroidectomized rats rendered moderately hyperthyroidic by daily infusion of T_4_, suggesting that maternal hyperthyroidism may affect fetal neurodevelopment.

There are no data regarding associations between subclinical hyperthyroidism during pregnancy and maternal health, although clinical hyperthyroidism has been related to preeclampsia (Millar et al. 1994). It is also unclear whether thyroid dysfunction during pregnancy is related to pre- or postpartum TH status. In the nonpregnant state, however, depressed TSH suggests that a woman’s free T_4_ and/or T_3_ is above her own individual set point, which can be indicative of mild thyroid failure (Andersen et al. 2003). Studies conducted in nonpregnant adults report that subclinical hyperthyroidism may be associated with all-cause mortality, cardiovascular mortality, cardiac dysfunction, reduced bone mineral density, and increased fracture risk (Surks et al. 2004).

The present study has a number of strengths. We used state-of-the-art methods to measure TH, including equilibrium dialysis for free T_4_ and an ultrasensitive third-generation assay with low LODs to measure TSH. We also had information on a large number of potential confounders, including demographic characteristics and environmental exposures to other endocrine disruptors such as lead, PCBs, and organochlorine pesticides. In addition, these results were unchanged after the exclusion of outliers and were robust to the lipid-adjustment method and to the summation method for ∑PBDEs (weight or molar basis).

The strong correlation among PBDE congeners, however, hampered our ability to distinguish their independent association, and the cross-sectional nature of this study limits causal inference. Reverse causation, for instance, cannot be excluded because TH regulates a number of metabolic pathways, including lipid metabolism and the activity of some cytochrome P450 enzymes (Takahashi et al. 2010; Yen 2005), which may alter PBDE serum concentrations. In addition, the mechanism of action for reduced TSH has not been clearly established. Possibly because of their structural similarity with T_4_ and T_3_, hydroxylated PBDEs (OH-PBDEs) have been shown to bind to thyroid receptors α1 and β and may thus inhibit the release of TSH by the pituitary (Marsh et al. 1998). Exposure of human hepatocytes to BDE-99 *in vitro* has also been shown to up-regulate type I deiodinase, which is involved in the deiodination of T_4_ to T_3_ and reverse-T_3_ (Stapleton et al. 2009). Elevated T_3_ would result in decreased TSH levels, but we did not measure T_3_ in this study because of limited sample volume. In addition, other chemicals have been shown to lower TSH through binding to the retinoid X receptor or interference with neuroendocrine signaling pathways (Haugen 2009), but few studies have investigated whether PBDEs act through these mechanisms.

It is noteworthy that studies conducted in rodents generally reported a hypothyroxinemic effect of exposure to PBDEs whereas human studies suggest a hyperthyroidic effect. Discrepancies between human and animal studies may be due to the high doses used in animal studies and physiologic differences. For instance, OH-PBDEs have been shown to competitively bind to human transthyretin (TTR), possibly resulting in increased T_4_ clearance (Meerts et al. 2000). Although TTR binds 75% of the circulating T_4_ in rats (Chanoine et al. 1992), it only binds 10–15% in humans (Robbins 2000), and thus effects of PBDEs through this mechanism may be stronger in rats than in humans. Animal studies have also reported that the PBDE commercial mixtures DE-71, DE-79, and Bromkal 70-5DE induce UDP-GT (Hallgren et al. 2001; Zhou et al. 2001), which catalyzes the glucuronidation of T_4_, the rate-limiting step in T_4_ elimination. It is, however, unclear whether PBDEs induce UDP-GT in humans.

## Conclusion

We report an inverse association between TSH and ∑PBDEs and BDEs 28, 47, 99, 100, and 153 serum concentrations in pregnant women around the 27th week of gestation. Odds of subclinical hyperthyroidism were also elevated in association with increased exposure to some of these chemicals. We observed these findings in a population with median serum PBDE concentrations within the range of a nationally representative sample. Although maternal clinical hyperthyroidism has been associated with adverse pregnancy outcomes such as preeclampsia, premature births, and low birth weight, few data are available on the direct effects of maternal subclinical hyperthyroidism on fetal and child development. In future analyses, we thus intend to examine whether subclinical hyperthyroidism and maternal exposure to PBDEs are associated with these outcomes.

## Figures and Tables

**Table 1:** ∑PBDE serum concentrations (ng/g lipids) around the 27th week of gestation by demographic characteristics in a population of pregnant women participating in the CHAMACOS study (*n* = 270).

Characteristic	No. (%)^a^	Geometric mean (GSD)
Age (years)		
18–24	129 (48)	27.3 (2.9)
25–29	90 (33)	23.8 (2.5)
30–34	34 (13)	27.2 (2.7)
35–45	17 (6)	35.6 (2.7)

Race/ethnicity		
Caucasian	7 (3)	84.1 (4.3)**
Latino	257 (95)	25.3 (2.6)
Other	6 (2)	54.3 (2.8)

Education		
≤ 6th grade	110 (41)	20.8 (2.3)#
7–12th grade	97 (36)	26.8 (2.8)
≥ High school diploma	63 (23)	39.5 (2.9)

Family income		
≤ Poverty line	156 (62)	25.1 (2.5)
Poverty line to 200%	85 (34)	31.7 (3.1)
> 200%	10 (4)	17.7 (2.1)

Country of birth		
United States	37 (14)	57.9 (3.0)#
Mexico	226 (84)	23.3 (2.5)
Other	7 (3)	30.0 (3.5)

Time in the United States (years)		
≤ 5	146 (54)	21.5 (2.7)#
6–10	57 (21)	27.1 (2.2)
≥ 11	67 (25)	41.4 (2.7)

Parity		
0	89 (33)	23.7 (2.9)
≥ 1	181 (67)	28.1 (2.6)

Smoking during pregnancy		
Yes	19 (7)	35.2 (3.0)
No	251 (93)	26.0 (2.7)

Alcohol drinking during pregnancy		
Yes	62 (24)	30.3 (2.9)
No	199 (76)	26.1 (2.7)

Drug use during pregnancy		
Yes	4 (2)	86.3 (2.3)*
No	256 (98)	26.4 (2.7)

Prepregnancy body mass index		
< 25	101 (39)	23.2 (2.5)
25–30	102 (40)	30.0 (3.0)
> 30	54 (21)	29.1 (2.6)

GSD, geometric standard deviation.

a Frequencies may not add to the total number of participants because of missing values. Percentages may not add to 100% because of rounding.

* *p* < 0.05.

** *p* < 0.01.

# *p* < 0.001 (two-sided *p*-values using ANOVA).

**Table 2:** PBDE congeners serum concentration (ng/g lipids) around the 27th week of pregnancy in a population of pregnant women participating in the CHAMACOS study.

PBDEs	*n*	LOD range	Percent detection	GM	95% CI	Min	25th percentile	Median	75th percentile	Max
∑PBDEs^a^	270	0.2–2.6	100.0^b^	26.5	25.0–31.5	3.6	13.1	25.2	42.3	1338.6
BDE-17	268	0.2–0.7	1.9	—	—	< LOD	< LOD	< LOD	< LOD	2.8
BDE-28	268	0.2–0.7	52.2	0.6	0.5–0.7	< LOD	< LOD	0.5	1.5	29.7
BDE-47	270	0.8–2.6	99.6	15.3	13.5–17.3	< LOD	7.7	15.0	25.8	761.0
BDE-66	268	0.2–0.7	14.9	—	—	< LOD	< LOD	< LOD	< LOD	10.1
BDE-85	270	0.2–0.7	47.8	—	—	< LOD	< LOD	< LOD	0.6	27.4
BDE-99	270	0.2–0.7	99.6	4.5	3.9–5.1	< LOD	2.3	4.0	6.7	298.0
BDE-100	270	0.2–0.7	98.5	2.8	2.5–3.1	< LOD	1.5	2.4	4.2	138.0
BDE-153	270	0.2–0.7	98.5	2.4	2.1–2.7	< LOD	1.3	2.1	3.8	96.9
BDE-154	270	0.2–0.7	41.9	—	—	< LOD	< LOD	< LOD	0.6	20.6
BDE-183	270	0.2–0.7	30.6	—	—	< LOD	< LOD	< LOD	0.4	5.9

Abbreviations: GM, geometric mean; Max, maximum; Min, minimum. We did not calculate geometric means and their respective 95% CIs for congeners with detection frequencies < 50% (—).

a Sum of congeners with detection frequencies > 50% (BDEs 28, 47, 99, 100, and 153).

b Percentage of samples with at least one congener above the LOD.

**Table 3:** Associations between PBDE and TH serum concentrations in pregnant women participating in the CHAMACOS study.

	Free T_4_ (ng/dL)				Total T_4_ (μg/dL)				Log_10_ TSH (mIU/L)			
	Unadjusted		Adjusted^a^		Unadjusted		Adjusted^a^		Unadjusted		Adjusted^a^	
PBDE	β (95% CI)	*R*^2^	β (95% CI)	*R*^2^	β (95% CI)	*R*^2^	β (95% CI)	*R*^2^	β (95% CI)	*R*^2^	β (95% CI)	*R*^2^
∑PBDEs	0.01 (−0.06 to 0.07)	< 0.01	0.02 (−0.05 to 0.09)	0.07	−0.06 (−0.51 to 0.40)	< 0.01	−0.18 (−0.65 to 0.30)	0.09	−0.07 (−0.14 to −0.01)*	0.02	−0.08 (−0.14 to −0.01)*	0.10

BDE-28	0.00 (−0.05 to 0.05)	< 0.01	0.01 (−0.05 to 0.06)	0.07	0.12 (−0.23 to 0.47)	< 0.01	0.07 (−0.28 to 0.42)	0.09	−0.05 (−0.10 to −0.01)*	0.02	−0.05 (−0.10 to 0.00)*	0.10

BDE-47	0.00 (−0.07 to 0.06)	< 0.01	0.01 (−0.06 to 0.08)	0.07	−0.05 (−0.48 to 0.39)	< 0.01	−0.15 (−0.60 to 0.30)	0.09	−0.07 (−0.13 to −0.01)*	0.02	−0.07 (−0.13 to −0.01)*	0.10

BDE-99	−0.01 (−0.08 to 0.06)	< 0.01	0.00 (−0.07 to 0.07)	0.07	−0.05 (−0.49 to 0.39)	< 0.01	−0.18 (−0.62 to 0.27)	0.09	−0.06 (−0.12 to 0.00)#	0.01	−0.07 (−0.13 to 0.00)*	0.10

BDE-100	−0.01 (−0.07 to 0.06)	< 0.01	0.01 (−0.06 to 0.08)	0.07	−0.03 (−0.48 to 0.42)	< 0.01	−0.11 (−0.58 to 0.36)	0.09	−0.09 (−0.15 to −0.02)**	0.03	−0.09 (−0.15 to −0.02)**	0.10

BDE-153	0.04 (−0.04 to 0.11)	< 0.01	0.06 (−0.02 to 0.14)	0.08	−0.15 (−0.63 to 0.33)	< 0.01	−0.27 (−0.79 to 0.25)	0.09	−0.08 (−0.14 to −0.01)*	0.02	−0.08 (−0.15 to −0.01)*	0.10

PBDE serum concentrations were log_10_-transformed.

a Adjusted for maternal age at enrollment, education, country of birth, gestational age at the time of blood collection, and family income as well as maternal HCB and PCB serum concentrations.

* *p* < 0.05.

** *p* < 0.01.

# *p* < 0.10.

**Table 4:** Adjusted ORs (95% CIs) for subclinical hyperthyroidism in relation with PBDE serum concentrations (ng/g lipids) in pregnant women participating in the CHAMACOS study.^a^

PBDE	Continuous^b^	Quartile 2^c^	Quartile 3^c^	Quartile 4^c^
∑PBDEs	1.9 (0.8–4.5)	2.0 (0.7–6.3)	1.5 (0.5–4.8)	3.3 (1.0–10.3)*,##
BDE-28	1.6 (0.8–3.3)	0.5 (0.2–1.8)	1.0 (0.3–2.9)	2.3 (0.8–6.4)*
BDE-47	1.8 (0.8–4.1)	1.7 (0.6–5.0)	1.3 (0.4–4.0)	2.3 (0.8–7.0)*
BDE-99	1.6 (0.7–3.7)	1.1 (0.4–3.3)	1.1 (0.3–3.3)	1.9 (0.7–5.5)
BDE-100	2.1 (0.9–4.9)*	3.2 (1.0–10.4)#	1.9 (0.5–6.5)	3.9 (1.2–12.9)*,##
BDE-153	2.4 (0.9–6.1)*	3.0 (0.9–9.8)#	3.7 (1.2–11.6)##	3.2 (1.0–13.9)**,##

a Adjusted for maternal age at enrollment, education, country of birth, gestational age at the time of blood collection, and family income as well as maternal HCB and PCB serum concentrations.

b PBDE serum concentrations were log_10_-transformed.

c ORs relative to the first quartile of PBDE serum concentration.

* *p* < 0.10.

** *p* < 0.05 on tests for linear trends for continuous or categorical PBDEs.

# *p* < 0.10.

## *p* < 0.05 relative to quartile 1.
